# LAPAROSCOPIC ANTIREFLUX SURGERY IN PATIENTS WITH EXTRA ESOPHAGEAL SYMPTOMS RELATED TO ASTHMA

**DOI:** 10.1590/S0102-67202014000200002

**Published:** 2014

**Authors:** Amanda Pinter Carvalheiro da SILVA, Valdir TERCIOTI-JUNIOR, Luiz Roberto LOPES, João de Souza COELHO-NETO, Laura BERTANHA, Paulo Rodrigo de Faria RODRIGUES, Nelson Adami ANDREOLLO

**Affiliations:** Disciplina de Moléstias do Aparelho Digestivo do Departamento de Cirurgia e Gastrocentro da Faculdade de Ciências Médicas da Universidade Estadual de Campinas, Unicamp (Digestive Diseases Surgical Unit, Department of Surgery and Gastrocenter, School of Medical Sciences, State University of Campinas, UNICAMP), Campinas, SP, Brazil.

**Keywords:** Refluxo gastroesofágico, Asma, Videolaparoscopia, Fundoplicatura

## Abstract

**Background:**

Asthma, laryngitis and chronic cough are atypical symptoms of the gastroesophageal reflux disease.

**Aim:**

To analyze the efficacy of laparoscopic surgery in the remission of extra-esophageal symptoms in patients with gastroesophageal reflux, related to asthma.

**Methods:**

Were reviewed the medical records of 400 patients with gastroesophageal reflux disease submitted to laparoscopic Nissen fundoplication from 1994 to 2006, and identified 30 patients with extra-esophageal symptoms related to asthma. The variables considered were: gender, age, gastroesophageal symptoms (heartburn, acid reflux and dysphagia), time of reflux disease, treatment with proton pump inhibitor, use of specific medications, treatment and evolution, number of attacks and degree of esophagitis. Data were subjected to statistical analysis, comparing the pre- and post-surgical findings.

**Results:**

The comparative analysis before surgery (T1) and six months after surgery (T2) showed a significant reduction on heartburn and reflux symptoms. Apart from that, there was a significant difference between the patients with daily crises of asthma (T1 versus T2, 45.83% to 16.67%, p=0.0002) and continuous crises (T1, 41.67% versus T2, 8.33%, p=0.0002).

**Conclusion:**

Laparoscopic Nissen fundoplication was effective in improving symptoms that are typical of reflux disease and clinical manifestations of asthma.

## Abstract (pt)

**Racional:**

A asma, a laringite e a tosse crônica são sintomas atípicos da doença do refluxo gastroesofágico.

**Objetivo:**

Analisar a eficácia da cirurgia laparoscópica na remissão de sintomas extra-esofágicos em doentes com refluxo gastroesofágico, relacionada com a asma.

**Métodos:**

Foram revisados os prontuários de 400 doentes com doença do refluxo gastroesofágico submetidos à fundoplicatura a Nissen entre 1994 e 2006 e foram identificados 30 casos com sintomas extra-esofágicos relacionadas à asma. As variáveis consideradas foram: sexo, idade, sintomas gastroesofágicos (azia, refluxo ácido e disfagia), o tempo da doença do refluxo, o tratamento com inibidores da bomba de prótons, o uso de medicamentos específicos, tratamento e a evolução, o número de crises e o grau de esofagite. Os dados foram submetidos à análise estatística, comparando os resultados pré e pós-operatórios.

**Resultados:**

A análise comparativa antes da operação (T1) e seis meses após (T2) mostrou redução significativa dos sintomas de azia e refluxo. Além disso, houve diferença significativa entre os doentes com crises diárias de asma (T1, versus T2, 45,83% para 16,67%, p=0,0002) e crises contínuas (T1, 41,67% versus T2, 8,33%, p=0,0002).

**Conclusão:**

A fundoplicatura à Nissen por via laparoscópica foi eficaz na melhora dos sintomas que são típicos da doença do refluxo e manifestações clínicas da asma, melhorando a qualidade de vida.

## INTRODUCTION

The clinical manifestations that are considered typical symptoms of the gastroesophageal reflux disease (GERD) are mainly heartburn, regurgitation and dysphagia^1^. Atypical and extra-esophageal symptoms are asthma, bronchitis, idiopathic pulmonary fibrosis, hoarseness, subglottic stenosis, granuloma of the vocal fold and laryngeal carcinoma and other extra-esophageal manifestations such as non-cardiac chest pain, sinusitis, pharyngitis and apnea sleep^2^. The atypical symptoms of reflux disease can occur in up to 74.4% of the patients with GERD^3,4^.

The upper digestive endoscopy and contrast radiography of the esophagus, stomach and duodenum may be useful in the diagnosis of GERD and have been an important tool for the detection of esophageal complications. The 24 h-pH monitoring plays an important role in the evaluation of patients with extra-esophageal manifestations. However, a positive test only confirms the coexistence between pathologic gastroesophageal reflux and symptoms, not ensuring a relationship of cause and effect^2^.

Asthma is considered the main extra-esophageal manifestation of GERD. Among the drugs used for asthma control, some may favor reflux because they relax the smooth muscles of the esophagus and stomach, such as theophylline and beta-adrenergic agonists, causing a delay in gastric emptying, which creates a pro-reflux effect. The clinical treatment of GERD collaborates in controlling symptoms of asthma, with the possibility of reducing medication, although without satisfactory changes in the respiratory parameters^2,4^.

The antireflux surgery has been shown to significantly improve respiratory symptoms associated with GERD and reduce the need for medications. This fact has a great importance since the atypical manifestations require more aggressive antisecretory therapy when compared with typical GERD symptoms. The association between atypical symptoms and GERD is still not so clear and there is some controversy about the effectiveness treating these symptoms. Thus, it is justified to investigate the prevalence of symptoms and control analysis of these symptoms with surgical treatment, using laparoscopic fundoplication^2^.

The aim of this study is to analyze the effectiveness of laparoscopic fundoplication in the remission of extra-esophageal symptoms of asthma in patients with GERD.

## METHODS

Were reviewed the medical records of 400 patients treated in the Unicamp University Hospital, Campinas, SP, Brazil, with confirmed diagnosis of gastroesophageal reflux disease, aged over 18 years old, and who underwent laparoscopic fundoplication from 1994 and 2006; were identified 30 patients with extra-esophageal symptoms related to asthma (7,5%).

The classification of asthma used was the recommended by the Guidelines of the Brazilian Medical Association and the Federal Council of Medicine^5^. The variables considered were: gender, age, gastroesophageal symptoms (heartburn, acid reflux and dysphagia), time history of reflux disease, treatment with proton pump inhibitor, use of specific medications, symptoms, treatment and evolution, number of recurrences, degree of esophagitis and data from additional tests.

All patients presented preoperative radiological examinations showing gastroesophageal reflux and upper digestive endoscopy pre- and postoperative.

Were compared the data from the patients before (Time 1 - T1) and six months after (Time 2 - T2) the laparoscopic surgery, considering the following parameters: the intensity of the gastroesophageal symptoms according to the use and dosage of the specific medication, the number of asthma crisis and degree of esophagitis. The last interview of the patients evaluated after surgery was considered the Time 3 - T3, which happened on average at 37 months follow-up.

The surgical technique employed was the laparoscopic Nissen fundoplication (short and flopping)^2^ performed by the same surgeons team.

The asthma crisis were classified as intermittent (none or <2/week), mild (3-4/week), moderate (daily) and severe (continuous). The degree of esophagitis was obtained by upper endoscopy and data were classified, in degrees, by the Los Angeles classification.

Data were subjected to statistical analysis, comparing the pre- and postoperative findings. For this, was used the SAS program (Statistical Analysis System, SAS Institute Inc., 1996).

To describe the profile of the studied group, according to the previously established variables, frequency tables were made for categorical variables and arithmetic descriptive statistics of the continuous variables. The comparison of proportions was performed using the chi-square or Fisher's exact test, when necessary. Continuous variables were compared by Student's t test for independent samples. In all cases, was used a significance level of 5% (p<0.05) to reject the null hypothesis.

## RESULTS

Among the 30 patients included in the study, 17 (58.62%) had reflux disease for more than five years, while 29 (96.67%) had heartburn, 21 (70%) had reflux and 15 (50%) mild dysphagia. Regarding the treatment during this period, 16 (53.33%) used H2-receptor antagonist and 24 (80%), proton pump inhibitor. Using the Los Angeles classification to assess the degree of esophagitis, 10 (33.33%) were in class A, five (16.67%) in class B, four (13.33%) in class C, five (16.67%) in class D and the remaining six (20%) had no esophagitis.

The comparative analysis between the pre-operative evaluation (T1) and six months after surgery (T2) showed a significant reduction in symptoms of heartburn, reflux and the use of proton pump inhibitors and H2 receptor antagonist. On the other hand, the symptom of dysphagia showed a non-significant reduction (Table 1).

**TABLE 1:** Questions concerning hernia relapse (1 and 2)

Variables	T1	T2	p
Heartburn	25 (96.15%)	5 (19.23%)	<0.0001(*)
Reflux	19 (73.08%)	3 (11.54%)	<0.0001(*)
Disphagia	13 (50%)	6 (23.08%)	0.0522
Use of proton pump inhibitor	20 (80%)	2 (8%)	<0.0001(*)
Use of H2-receptor antagonist	14 (56%)	2 (8%)	0.0013(*)

* p<0.05

After six months of surgery (T2), it was observed a significant improve in the degree of the esophagitis, using the Los Angeles Classification (Table 2).

**TABLE 2:** Comparison of the degree of esophagitis between times 1 and 2

Degree of esophagitis	T1	T2 (*)
No esophagitis	2 (15.38%)	2 (15.38%)
A	6 (46.15%)	2 (15.38%)
B	2 (15.38%)	0 (0.00%)
C	2 (15.38%)	1 (7.69|%)
D	1 (7.69|%)	1 (7.69|%)

* p=0.0038 – p<0,05

The preoperative data showed that 27 patients (90%) had asthma crisis for over five years, being that 10% had less than two asthma crisis per week, 10% had 3-4 per week, 40% had daily crises and 40% had continuing crises.

Comparing T1 and T2, there was a significant difference between the patients with daily asthma crises (T1, 45.83% versus T2, 16.67%, p=0.0002) and continuous crises (T1, 41.67% versus T2, 8,33%, p=0.0002). Table 3 shows the data of the clinical interview about hospitalization for asthma, limitation of physical activity and missed work days, and use of mechanical ventilation before surgery (T1) and after the surgery (T3).

**TABLE 3:** Comparative analysis of parameters of asthma severity

Variable	T1	T3	p
Hospitalization for asthma	17 (73.91%)	3 (13.04%)	0.0002(*)
Limitation of physical activity	20 (86.96%)	3 (13.04%)	<0.0001(*)
Absence from work	15 (65.22%)	2 (8.70%)	0.0003(*)
Need for mechanical ventilation	7 (30.43%)	1 (4.35%)	0.0143(*)

* p<0.05

Regarding the medication used to control asthma, there was no significant difference between T1 and T2 in the use of short-acting beta agonist, xanthine, anticholinergics, inhaled corticosteroids and long-term beta agonist; however, there was a significant reduction in the use of oral steroid therapy (Table 4).

**TABLE 4:** Comparative analysis of medication related to atypical symptoms between times 1 and 2

Variables	T1	T2	p
Short-acting beta agonist	21 (91.30%)	18 (78.26%)	0.2568
Anticholinergic	12 (52.17%)	7 (30.43%)	0.1317
Oral corticosteroids	12 (52.17%)	5 (21.74%)	0.0082(*)
Xantine	12 (52.17%)	9 (39.13%)	0.1797
Long-acting beta agonist	9 (39.13%)	8 (39.78%)	0.6547
Inhaled corticosteroids	15 (65.22%)	14 (60.87%)	0.7389

(*) p<0.05

Comparing T1 with the last postoperative assessment (T3), there was a significant difference in the use of short-acting beta agonist, xanthine, long-term beta agonist and the reduction of oral steroid therapy remained significant (Table 5).

**TABLE 5:** Comparative analysis of medication related to atypical symptoms between times 1 and 3

Variables	T1	T2	p
Short-acting beta agonist	21 (91.30%)	11 (47.83%)	0.0039 (*)
Anticholinergic	12 (52.17%)	5 (21.74%)	0.0348(*)
Oral corticosteroids	12 (52.17%)	3 (13.04%)	0.0027(*)
Xantine	12 (52.17%)	4 (17.39%)	0.0047(*)
Long-acting beta agonist	9 (39.13%)	18 (78.26%)	0.0067(*)
Inhaled corticosteroids	15 (65.22%)	16 (69.57%)	0.7389

* p<0.05

Comparing T2 to T3, most parameters showed no significant differences, only the symptom of dysphagia increased T3 (T2, 23.08% versus T3, 57.69%, p=0.0067), the use of short-acting beta agonist was reduced (T2, 78.26% versus T3, 47.83%, p= 0.0196) and the use of long-acting beta agonists increased (T2, 34.78% versus T3, 78.26%, p=0.0039).

## DISCUSSION

The GERD is secondary the reduced efficacy of esophageal anti-reflux mechanisms, especially the tone of the lower esophageal sphincter, sliding hiatal hernia, slow or inadequate elimination of the refluxate, slow gastric emptying time, and increased gastric volume^1^.

The treatment of reflux disease is mainly clinical, with the use of antacids and acid blockers. Failure to control the symptoms with medication suggests misdiagnosis or relatively serious ailment. In this study, 80% of the patients were using proton pump inhibitor and 53.33%, the H2 receptor antagonists during pre-operatory, without symptomatic control. Moreover, the majority of the patients (58.62%) had the reflux disease for over five years, a factor that contributes to the ineffectiveness of the drug therapy, as it is shown in the literature that more than 50% of patients with chronic reflux have frequent recurrence of symptoms despite appropriate treatment^3,6^.

Although the aim of the medical and surgical treatment is to relieve the symptoms, surgery also generates a mechanical solution, while drug therapy is limited to acid suppression^7^.

The laparoscopic antireflux fundoplication allows the maintenance of the gastroesophageal junction in intra-abdominal position and construction of a valve with the gastric fundus, re-safely establishing the competence of the lower esophageal sphincter through the mechanical improvement of its function^2,8^.

The anti-reflux surgery has been very effective in relieving symptoms in 88-95% of patients with good patient satisfaction in studies in the short and long term. These results include patients with complicated GERD, large hiatal hernias, refractory esophagitis and peptic stricture^6,8^.

This study shows that there was a significant improvement in symptoms of heartburn (T1, 96.15% versus T2, 19.23%, p<0.0001) and reflux (T1, 73.08% versus T2, 11.54%, p<0.0001), with no statistical difference between T2 and T3, showing that the surgery was effective in controlling typical symptoms.

Recently, other authors recorded that surgery has been proven effective, but there are doubts about the durability of the action. Long-term results showed that after 10 years, 92% of the patients randomized to the medication continued its use and 62% of those who were initially treated with surgery returned to use drug therapy^8,9,10^. In this study, few patients maintained use of antacids postoperatively. The use of proton pump inhibitors and H2 receptor antagonist decreased (T1, 80% versus T2, 8%, p<0.0001) and (T1, 56% versus T2, 8%, p=0.0013), respectively. Furthermore, was observed no significant difference in the use of proton pump inhibitors or H2 receptor blockers between times T2 and T3.

Dysphagia is one of the most common side effects of laparoscopic antireflux surgery in the immediate postoperative period, usually improving the first six weeks after surgery. The persistent dysphagia may occur in 2-5% of the patients^2,4,11^. In this study 15 (50%) of the patients already had symptoms in the preoperative evaluation, with no significant reduction in the postoperative period of approximately six months (T1, 50% versus T2, 23.08%, p=0.0522), probably due to esophageal dismotility. The fact that the symptom of dysphagia increased in T3 (T2, 23.08% versus T3, 57.69%, p=0.0067) was not expected.

Most of the patients presented with esophagitis before surgery, and six months after 69.23% (p=0.0038) of the sample not presenting this complication. In relation to the effects of acid-reduction therapy in asthma control, some authors reviewed 12 studies and the pooled data showed an improvement in asthma symptoms in 69% of patients, and 62% reduced bronchodilator use without increased exacerbations^8,10^.

Another group of researchers studied respiratory symptoms in GERD patients initially treated with proton pump inhibitor and subsequently with anti-reflux surgery and found that after six months of treatment with proton pump inhibitor, the respiratory symptoms decreased in only 14% of the patients and no patient remained asymptomatic during this period. After laparoscopic fundoplication, the decrease in respiratory symptoms occurred in 86% of the patients and 67% remained asymptomatic until the end of the study ^8,9,10,12,13^.

This study showed, between periods T1 and T2, the reduction in oral steroid therapy (T1, 52.17% versus T2, 21.74%, p=0.0082) with a significant difference. Lately, there was a decrease in the use of short-acting beta agonist (T1, 91.30% versus T3, 47.83%, p=0.0039), xanthine (T1, 52.17% versus T3, 17.39%; p=0.0047) and the use of oral corticosteroids (T1, 52.17% versus T3, 13.04%, p=0.0027), as well as an increased use of long-acting beta agonist (T1, 39.13% versus T3, 78.26%, p=0.0067). These data suggest decreased asthma crisis, lower severity of the crisis and higher use of such maintenance therapeutic versus treatment of crises, which may be interpreted as a clinical improvement of the patient.

Sontag et al. analyzed 62 patients with both GERD and asthma, in a randomized study, divided in three types of treatments: control group (24 cases), treatment of reflux with ranitidine 150 mg three times a day (22 cases) and surgical treatment with Nissen fundoplication (16 cases). After a 2-year follow-up, 75% of surgical patients had improvement in nocturnal asthma exacerbations, compared with 9.1% and 4.2% of patients on medical therapy and controls, respectively^14^.

Westcott et al. studied 41 patients with documented laryngopharyngeal reflux disease and symptoms of hoarseness, vocal fatigue, globus, chronic throat clearing, chronic cough, and laryngospasm submitted to laparoscopic floppy Nissen fundoplication. They concluded that the laryngeal symptoms secondary to GERD after fundoplication and may take longer to be resolved compared to esophageal symptoms, however, a subjective overall response rate of 85% positive is possible^15^.

Koch et al. analyzed the results of partial (Toupet, n=50) and total fundoplication (floppy Nissen, n=50) in a hundred patients with extraesophageal symptoms (cough, asthma, hoarseness, and distortion of taste) in a selected cohort of patients with GERD, using a standardized symptom questionnaire before surgery and at three and 12 month follow-up.They concluded that the laparoscopic fundoplication is justified for patients with documented GERD and atypical symptoms refractory to medical treatment. However, the Toupet fundoplication may have a lesser effect on asthma^16^.

In agreement with the literature, was observed a significant differences between the patients with daily symptoms of asthma (T1, 45.83% versus T2, 16.67%, p= 0.0002) and persistent symptoms (T1, 41.67% and T2, 8.33%, p=0.0002), moreover, this reduction was maintained, since there was no significant difference between T2 and T3 (p=0.2827, p>0.05).

The data analyzed from the clinical interview are noteworthy, where prior to surgery, 17 patients (73.91%) reported hospitalization for asthma, also with the need for mechanical ventilation in seven (30.43%) patients. In the postoperative data, only tgree (13.04%, p=0.0002) were hospitalized and one (4.35%, p 0.0143) subjected to ventilatory support.

As for physical activity, 20 patients (86.96%) reported limitations before surgery, and 15 (65.22%) had the need to miss work. In the postoperative period, three (​​13.04%, p<0.0001) continued to show limitation of physical activity and two (8.70%, p=0.0003) had to miss work.

## CONCLUSION

The laparoscopic Nissen fundoplication was effective in decreasing the symptoms that are typical of reflux disease and clinical manifestations of asthma, allowing the reduction of the drugs employed during the studied period, and improving the quality of life.
